# Supplementary material for: The effect of hearing aids on cognitive function: A systematic review
Source: PLoS One. 2021 Dec 31;16(12):e0261207. doi: 10.1371/journal.pone.0261207 (PMC8719768; doi:10.1371/journal.pone.0261207)
Supplement: S1 File — (DOCX) [file pone.0261207.s001.docx]

**Appendix1. Search String**

**PubMed**

(Hearing aid*[MeSH Terms] OR Hearing aid*[Title/Abstract] OR Hearing-aid*[Title/Abstract] OR Hearing instrument*[Title/Abstract] OR Hearing device*[Title/Abstract] OR Ear mold*[Title/Abstract] OR hearing amplif*[Title/Abstract])

AND

(Cognit*[MeSH Terms] OR Cognit*[Title/Abstract] OR Cognition disorders[MesH Terms] OR Cognitive function*[Title/Abstract] OR Memory[MeSH Terms] OR Memor*[Title/Abstract] OR Attention[MeSH Terms] OR Attention[Title/Abstract] OR (Dementia[Mesh] OR Dement*[Title/Abstract] OR Executive function*[MeSH Terms]) OR Executive function*[Title/Abstract])

**Embase**

 (‘hearing aid'/exp OR ‘Hearing aid’:ti,ab,kw OR ‘Hearing-aid’:ti,ab,kw OR ‘Hearing instrument’:ti,ab,kw OR ‘Hearing device’:ti,ab,kw OR ‘Ear mold’:ti,ab,kw OR ‘hearing amplify’:ti,ab,kw)

AND

('cognition'/exp OR 'memory'/exp OR 'attention'/exp OR 'executive function'/exp OR 'dementia'/exp OR ‘Cognit*’:ti,ab,kw OR ‘Cognitive function’:ti,ab,kw OR ‘Memor*’:ti,ab,kw OR ‘Attention’:ti,ab,kw OR ‘Dement’:ti,ab,kw OR ‘Executive function’:ti,ab,kw)

**Cochrane**

MeSH descriptor: [Hearing Aids] explode all trees

OR (Hearing aid):ti,ab,kw OR (Hearing-aid):ti,ab,kw OR (Hearing instrument):ti,ab,kw OR (Hearing device):ti,ab,kw OR (Ear mold):ti,ab,kw OR (hearing amplify):ti,ab,kw)

AND

MeSH descriptor: [Cognition] explode all trees

OR MeSH descriptor: [Cognition disorders] explode all trees

OR MeSH descriptor: [Memory] explode all trees

OR MeSH descriptor: [Attention] explode all trees

OR MeSH descriptor: [Executive function] explode all trees

OR MeSH descriptor: [Dementia] explode all trees

OR (Cognit*):ti,ab,kw OR (Cognitive function):ti,ab,kw OR (Memor*):ti,ab,kw OR (Attention):ti,ab,kw OR (Dement):ti,ab,kw OR (Executive function):ti,ab,kw)
