# Supplementary material for: The effect of hearing aids on cognitive function: A systematic review
Source: PLoS One. 2021 Dec 31;16(12):e0261207. doi: 10.1371/journal.pone.0261207 (PMC8719768; doi:10.1371/journal.pone.0261207)
Supplement: S2 File — (PDF) [file pone.0261207.s002.pdf]

National Institute for Health Research

University of York
Centre for Reviews and Dissemination

Systematic review

1. * Review title.

Give the title of the review in English

The effect of hearing aids on cognition: a systematic review

2. Original language title.

For reviews in languages other than English, give the title in the original language. This will be displayed with the English language title.

3. * Anticipated or actual start date.

Give the date the systematic review started or is expected to start.

16/02/2020

4. * Anticipated completion date.

Give the date by which the review is expected to be completed.

01/08/2020

5. * Stage of review at time of this submission.

Tick the boxes to show which review tasks have been started and which have been completed. Update this field each time any amendments are made to a published record.

Reviews that have started data extraction (at the time of initial submission) are not eligible for inclusion in PROSPERO. If there is later evidence that incorrect status and/or completion date has been supplied, the published PROSPERO record will be marked as retracted.

This field uses answers to initial screening questions. It cannot be edited until after registration.

The review has not yet started: No

Review stage	Started	Completed
Preliminary searches	Yes	No
Piloting of the study selection process	No	No
Formal screening of search results against eligibility criteria	No	No
Data extraction	No	No
Risk of bias (quality) assessment	No	No
Data analysis	No	No

Provide any other relevant information about the stage of the review here.

6. * Named contact.

The named contact is the guarantor for the accuracy of the information in the register record. This may be any member of the review team.

Maxime Sanders

Email salutation (e.g. "Dr Smith" or "Joanne") for correspondence:

Mr Sanders

7. * Named contact email.

Give the electronic email address of the named contact.

M.E.Sanders-7@umcutrecht.nl

8. Named contact address

Give the full institutional/organisational postal address for the named contact.

Department of otolaryngology-head and neck surgery, Heidelberglaan 100, 3584 CX Utrecht

9. Named contact phone number.

Give the telephone number for the named contact, including international dialling code.

+31 (0)88 755 66 44

10. * Organisational affiliation of the review.

Full title of the organisational affiliations for this review and website address if available. This field may be completed as 'None' if the review is not affiliated to any organisation.

UMC Utrecht. Department of otolaryngology-head and neck surgery

Organisation web address:

https://umcutrecht.nl

11. * Review team members and their organisational affiliations.

Give the personal details and the organisational affiliations of each member of the review team. Affiliation refers to groups or organisations to which review team members belong. **NOTE: email and country now MUST be entered for each person, unless you are amending a published record.**

Mr Maxime Sanders. UMC Utrecht

12. * Funding sources/sponsors.

Details of the individuals, organizations, groups, companies or other legal entities who have funded or sponsored the review.

None

Grant number(s)

State the funder, grant or award number and the date of award

13. * Conflicts of interest.

List actual or perceived conflicts of interest (financial or academic).

None

14. Collaborators.

Give the name and affiliation of any individuals or organisations who are working on the review but who are not listed as review team members. **NOTE: email and country must be completed for each person, unless you are amending a published record.**

15. * Review question.

State the review question(s) clearly and precisely. It may be appropriate to break very broad questions down into a series of related more specific questions. Questions may be framed or refined using PI(E)COS or similar where relevant.

What is the effect of hearing aids on cognitive function?

16. * Searches.

State the sources that will be searched (e.g. Medline). Give the search dates, and any restrictions (e.g. language or publication date). Do NOT enter the full search strategy (it may be provided as a link or attachment below.)

The search strategy will be applied to the following major biomedical bibliographic databases: PubMed, EMBASE, The Cochrane Library, as well as a Grey Literature search. The electronic search will be supplemented by the manual searches of the references from articles identified for inclusion or relevant review articles.

17. URL to search strategy.

Upload a file with your search strategy, or an example of a search strategy for a specific database, (including the keywords) in pdf or word format. In doing so you are consenting to the file being made publicly accessible. Or provide a URL or link to the strategy. Do NOT provide links to your search **results**.

The search terms “hearing aids” and “cognition” and all their synonyms were combined.

#1

Hearing aid[MeSH Terms]

OR Hearing aid*[Title/abstract]

OR Hearing-aid*[Title/Abstract]

OR Hearing device[Title/Abstract]

OR Hearing instrument*[Title/Abstract]

OR Ear mold*[Title/Abstract]

OR Hearing amplification[Title/Abstract]

#2

Cognition[MeSH Terms]

OR Cognit*[Title/Abstract]

OR Memory[MeSH Terms]

OR Memor*[Title/Abstract]

OR Attention[MeSH Terms]

OR Attention[Title/Abstract]

OR Dementia[MeSH Terms]

OR Dement*[Title/Abstract]

OR Executive function[MeSH Terms]

OR Executive function*[Title/Abstract]

#3

#1 AND #2

Alternatively, upload your search strategy to CRD in pdf format. Please note that by doing so you are consenting to the file being made publicly accessible.

Do not make this file publicly available until the review is complete

18. * Condition or domain being studied.

Give a short description of the disease, condition or healthcare domain being studied in your systematic review.

Patients with hearing loss starting treatment with hearing aids.

19. * Participants/population.

Specify the participants or populations being studied in the review. The preferred format includes details of both inclusion and exclusion criteria.

Inclusion criteria

- Studies on effect of hearing aids with cognitive function as an outcome
- Type: longitudinal

Exclusion criteria

- Animal studies
- Duplicate data sets
- Inability to calculate effect size
- Diagnosed dementia at onset of study

20. * Intervention(s), exposure(s).

Give full and clear descriptions or definitions of the interventions or the exposures to be reviewed. The

preferred format includes details of both inclusion and exclusion criteria.

Hearing aids.

21. * Comparator(s)/control.

Where relevant, give details of the alternatives against which the intervention/exposure will be compared (e.g. another intervention or a non-exposed control group). The preferred format includes details of both inclusion and exclusion criteria.

Usual care and/or no treatment.

22. * Types of study to be included.

Give details of the study designs (e.g. RCT) that are eligible for inclusion in the review. The preferred format includes both inclusion and exclusion criteria. If there are no restrictions on the types of study, this should be stated.

Longitudinal studies

23. Context.

Give summary details of the setting or other relevant characteristics, which help define the inclusion or exclusion criteria.

In 2015 it was estimated that 47 million people worldwide were living with dementia and as the world's population increases in age this number is expected to nearly triple by 2050. This is a huge burden on individuals with dementia and society as a whole. Economically, the WHO (2015) estimates that the total global societal cost of dementia is US$ 818 billion, equivalent to 1.1% of global domestic product . Dementia is a disorder characterized by cognitive decline involving one or more domains (learning and memory, language, executive function, complex attention, perceptual-motor, social cognition) interfering with daily function and independence . Mild cognitive impairment (MCI), an intermediate clinical state between normal cognition and dementia, is a mental state in which the extent of cognitive decline is greater than would be expected through aging and is often viewed as a precursor to dementia.

Originally dementia had been considered neither preventable nor treatable but paradigm shifts in thinking about dementia. In 2017 the Lancet Commissions on Dementia reported that 35% of risk factors are modifiable. Of these hearing loss is recognized as instigating 9% of dementia, the largest of the adjustable risk factors identified . Sensorineural hearing loss (SNHL) can successfully be treated with several types of hearing intervention, most commonly through conventional hearing aids. This systematic review will investigate the effect of hearing aids on cognitive function.

24. * Main outcome(s).

Give the pre-specified main (most important) outcomes of the review, including details of how the outcome is defined and measured and when these measurement are made, if these are part of the review inclusion criteria.

Cognitive function as measured by standardized assessments .

* Measures of effect

Please specify the effect measure(s) for you main outcome(s) e.g. relative risks, odds ratios, risk difference, and/or 'number needed to treat.

Relative risks, odds ratios, risk difference, mean difference.

25. * Additional outcome(s).

List the pre-specified additional outcomes of the review, with a similar level of detail to that required for main outcomes. Where there are no additional outcomes please state 'None' or 'Not applicable' as appropriate to the review

None

* Measures of effect

Please specify the effect measure(s) for you additional outcome(s) e.g. relative risks, odds ratios, risk difference, and/or 'number needed to treat.

None

26. * Data extraction (selection and coding).

Describe how studies will be selected for inclusion. State what data will be extracted or obtained. State how this will be done and recorded.

Study selection:

Two reviewers will independently screen all records by title and abstracts of retrieved studies and record their decisions. Studies describing the effect of hearing aids on cognitive function will be included according to the inclusion and exclusion criteria. Potentially relevant publications identified from the initial search and title and abstract screening will then undergo full-text screening by the two independent reviewers. Conflicts on the selection will be resolved by discussion.

Data extraction:

One reviewer will extract data and another one will check the extracted data. Study design, demographics, hearing level, follow-up duration and cognitive outcomes will be extracted. The studies will be examined by the review team for potential inclusion in a meta-analysis.

27. * Risk of bias (quality) assessment.

State which characteristics of the studies will be assessed and/or any formal risk of bias/quality assessment tools that will be used.

Two reviewers will independently assess risk of bias using the Cochrane Risk of Bias tool for randomized controlled trials and the ROBINS-I tool for non-randomized studies.

Disagreements between the reviewers will be resolved by discussion, with involvement of a third reviewer where necessary.

28. * Strategy for data synthesis.

Describe the methods you plan to use to synthesise data. This **must not be generic text** but should be **specific to your review** and describe how the proposed approach will be applied to your data. If meta-analysis is planned, describe the models to be used, methods to explore statistical heterogeneity, and software package to be used.

If sufficiently homogenous studies are found, a meta-analysis of aggregate cognitive function will be

conducted by pooling studies using fixed-effect and random-effects models with 95% confidence intervals. The heterogeneity of the included studies will guide the choice of model. Significant heterogeneity will necessitate a descriptive review of included studies. Authors will be contacted if additional data is needed.

29. * Analysis of subgroups or subsets.

State any planned investigation of 'subgroups'. Be clear and specific about which type of study or participant will be included in each group or covariate investigated. State the planned analytic approach.

Level of hearing loss, age.

30. * Type and method of review.

Select the type of review, review method and health area from the lists below.

Type of review

Cost effectiveness
No

Diagnostic
No

Epidemiologic
No

Individual patient data (IPD) meta-analysis
No

Intervention
Yes

Meta-analysis
Yes

Methodology
No

Narrative synthesis
Yes

Network meta-analysis
No

Pre-clinical
No

Prevention
No

Prognostic
No

Prospective meta-analysis (PMA)
No

Review of reviews
No

Service delivery
No

Synthesis of qualitative studies
No

Systematic review
Yes

Other
No

Health area of the review

Alcohol/substance misuse/abuse
No

Blood and immune system
No

Cancer
No

Cardiovascular
No

Care of the elderly
No

Child health
No

Complementary therapies
No

COVID-19
No

Crime and justice
No

Dental
No

Digestive system
No

Ear, nose and throat
Yes

Education
No

Endocrine and metabolic disorders
No

Eye disorders
No

General interest
No

Genetics
No

Health inequalities/health equity

No

Infections and infestations
No

International development
No

Mental health and behavioural conditions
No

Musculoskeletal
No

Neurological
Yes

Nursing
No

Obstetrics and gynaecology
No

Oral health
No

Palliative care
No

Perioperative care
No

Physiotherapy
No

Pregnancy and childbirth
No

Public health (including social determinants of health)
No

Rehabilitation
No

Respiratory disorders
No

Service delivery
No

Skin disorders
No

Social care
No

Surgery
No

Tropical Medicine
No

Urological
No

Wounds, injuries and accidents
No

Violence and abuse
No

31. Language.

Select each language individually to add it to the list below, use the bin icon to remove any added in error.
English

There is not an English language summary

32. * Country.

Select the country in which the review is being carried out. For multi-national collaborations select all the countries involved.

Netherlands

33. Other registration details.

Name any other organisation where the systematic review title or protocol is registered (e.g. Campbell, or The Joanna Briggs Institute) together with any unique identification number assigned by them. If extracted data will be stored and made available through a repository such as the Systematic Review Data Repository (SRDR), details and a link should be included here. If none, leave blank.

34. Reference and/or URL for published protocol.

If the protocol for this review is published provide details (authors, title and journal details, preferably in Vancouver format)

Add web link to the published protocol.

Or, upload your published protocol here in pdf format. Note that the upload will be publicly accessible.

No I do not make this file publicly available until the review is complete

Please note that the information required in the PROSPERO registration form must be completed in full even if access to a protocol is given.

35. Dissemination plans.

Do you intend to publish the review on completion?

Yes

Give brief details of plans for communicating review findings.?

36. Keywords.

Give words or phrases that best describe the review. Separate keywords with a semicolon or new line. Keywords help PROSPERO users find your review (keywords do not appear in the public record but are included in searches). Be as specific and precise as possible. Avoid acronyms and abbreviations unless these are in wide use.

Aging, cognition disorders, hearing loss, hearing aids, humans.

37. Details of any existing review of the same topic by the same authors.

If you are registering an update of an existing review give details of the earlier versions and include a full bibliographic reference, if available.

38. * Current review status.

Update review status when the review is completed and when it is published.New registrations must be ongoing so this field is not editable for initial submission.
Please provide anticipated publication date

Review_Ongoing

39. Any additional information.

Provide any other information relevant to the registration of this review.

40. Details of final report/publication(s) or preprints if available.

Leave empty until publication details are available OR you have a link to a preprint (NOTE: this field is not editable for initial submission). List authors, title and journal details preferably in Vancouver format.

Give the link to the published review or preprint.
