# Supplementary material for: The effect of hearing aids on cognitive function: A systematic review
Source: PLoS One. 2021 Dec 31;16(12):e0261207. doi: 10.1371/journal.pone.0261207 (PMC8719768; doi:10.1371/journal.pone.0261207)
Supplement: Attachment — Submitted filename: Response to Reviewers.docx [file pone.0261207.s004.docx]

Dear Editor,

We would like to thank the editor for the opportunity to resubmit the manuscript entitled ‘The effect of hearing aids on cognitive function: a systematic review’.

We received valuable comments of the reviewers of PlosOne and revised the paper carefully according to these comments to improve the quality of this manuscript. We would like to offer this improved manuscript to PlosOne for reconsideration of publication. Our detailed responses to the reviewers’ comments of the previous submission can be found below.

Thank you for considering our manuscript for publication.

Sincerely,

On behalf of the author-team,

I. Stegeman

| **Reviewer 1** | **Response** |
| --- | --- |
| This is a well written and interesting manuscript on an increasingly important topic. I have only minor comments below. I would be happy to review this paper again. | We thank the reviewer for this positive outlook towards our study and we appreciate the comments that will improve thepaper. |
|  |  |
| *Minor comments* |  |
| 48 – “described a model of life-course risk factors”. | We changed the text accordingly. Line: 50  *(*all linenumbers are based on the adjusted manuscript without marked changes)* |
| 50 – Hearing loss is estimated to account for up to 8.2% of dementia prevalence. Suggest making it clear that 8.2% come from 2020 update of Lancet Commission. Also, not clear why citation #6 is at end of sentence on line 51? | Thank you for hightlighting this unclarity. Text has been amended. Line: 52-53  Reference #6 instead of #5; reference mistake has been rectified. Line: 54 |
| 54 – third highest cause? | We changed the text accordingly. Line: 57 |
| 56 – suggest also including frequencies. | We added the frequencies. Line: 59-60 |
| 386 – should this be ‘describe an in-depth’. | Thank you for bringing this to our attention. Rectified. Line: 393 |
| 388 – common cause hypothesis refers to a common degenerative process effecting both the ear and brain. | It is a common degenerative process that affects sensory ability and intellectual functioning – due to a common factor, the deterioration of brain function. Therefore not the ear, but the auditory system, is affected. We changed this information in line 396 accordingly |
| 390 – ‘cognitive decline’. | We changed the text accordingly. Line: 397 |
| 391 – In reference to the sensory deprivation hypothesis, I would suggest avoiding the conclusion that cognitive decline would continue regardless of hearing aid use. This hypothesis refers to a more permanent or long/chronic change in comparison to the information-degradation hypothesis, but not necessarily completely permanent. Wayne et al (2015) is referenced here but also see Lindenberger et al (1994) and Humes et al (2013) (both referenced in Wayne). | We agree with the reviewer, when taking the other references in account that it is more considered to be a chronic change than a completely permanent change. We therefore have adjusted the information in line 397-398 accordingly |
| 415 – although. | Corrected. Line: 438 |
| 60/449 – suggest being consistent within the manuscript with the statistics referenced – i.e. use one, or both in both sentences. | We agree with the reviewer that we should be consistent with the statistics used and have adjusted the information in line 482-483 accordingly. |

| **Reviewer 2** | **Response** |
| --- | --- |
| Thank you for the opportunity to review this manuscript. The topic is relevant to clinicians and important in ongoing efforts to improve outcomes for people with dementia. Hearing loss is very common in this population, and mostly unaddressed. The paper has evaluated a range of studies meeting inclusion criteria. The review has been registered in PROSPERO and follows acceptable reporting guidelines. Risk of bias/quality assessment of included studies was undertaken. The authors may want to consider the following comments to improve their manuscript. | We thank the reviewer for their comments and also firmly believe in the importance of researching hearing loss, particularly in the population with dementia.  We have tried to address the comments and hope that this improves the clarity and flow of the paper. |
| Several reviews have already been done on this topic – how is this review different? The authors need to clearly spell out what new information their review offers. It is not obvious. | We agree with the reviewer that this issue is not stated clearing in the introduction, yet we did try to address this in the discussion. What is different about this systematic review, is that we have focused on longitudinal studies with participants without dementia, whom were first time users of hearing aids. Additionally, we offer a new perspective by examining the effects of hearing aids on the different cognitive domains as defined by the DSM-V criteria. We elaborated on this information in the discussion to clearly show the new information of this review. Line: 375-380 |
| This focusses on the clinical syndrome but makes no mention of the heterogeneity of the underlying disease pathology that may impact on the impact of HA on cognition. The range of dementia sub-types should be addressed, with specific reference to those which may impact on central auditory pathways | We agree that it would be very interesting to address the range of dementia sub-types. However, the studies included did not stratify dementia sub-types as outcomes. Considering the often relatively small scale of the studies, they would also have been underpowered to analyse this. Rightfully, this is the next step in terms of scientific research and a limitation to our knowledge currently and a limitation of this paper. Line: 457-462 |
| The introduction focuses on dementia, yet the review is about the impact of HA on cognition in adults without dementia. Hence, the introduction should downplay the dementia emphasis, or highlight ‘dementia prevention’ from the outset, including the lack of disease modifying therapies, rather than focusing on prevalence issues. | We thank the reviwer for their input. Our research question focused on cognitive decline as a whole and not purely on dementia. To highlight the societal and economic burden we choose to place a certain amount of emphasis on the prevalence of dementia. We however agree that our introduction should make this distinction clearer and have adjusted it accordingly. |
| It should be stated at the outset that this was only a narrative review and that a meta-analysis was not performed. This should be mentioned in the abstract and title as well to indicate the strength of the findings. | We thank the reviewer for their input and agree that we could more clearly state that this paper describing a systematic review, following the PRISMA guideline principes, is not a meta-analysis. In the abstract we added the information that a meta-analysis was not performed. Line 29-31 |
| A general concern is that the research question addresses the impact of HA use on cognition in any adult without dementia. The question is flawed. Instead, the question of interest is the impact of HA use on cognition or risk of dementia in people at risk of dementia. These group potentially follows a different cognitive decline trajectory than those not at risk, thus, the impact (and likely, the mechanism of action of HA) may differ. This needs to be discussed as a limitation. | This is an interesting discussion point that the reviewer addresses, especially when considering the wide variations in cognitive decline and dementia as a disease. What may have caused confusion is that in the introduction we put too much emphasis on dementia; herein sometimes using dementia and cognitive decline interchangibly. This has now been altered. In the end our research question systematically reviews the literature on prospective sutides examining the intervention effects of hearing aids and their effect on cognitive function in adults who have not been diagnosed with dementia. We therefore do not make a distinction between those more and less at risk of the disease of dementia and therefore do not feel this is a limitation to our study. In the future it would be interesting to see if there is a difference between these groups. |
|  |  |
| Minor comments |  |
| 60. The term ‘massively underutilized’ is slightly colloquial - consider ‘significantly underutilized’ instead. | We changed the text. Line: 63 |
| 70. This sentence is incomplete: ‘Especially considering that hearing aids are a relatively low-cost intervention when compared to the high societal and psychosocial costs that accompany declining cognitive ability’…?? | Thank you for noting this inconsistency. It has been adjusted. Line: 67-68 |
| Line 98 and 106, should read ‘the date WERE extracted’ (data is plural). [Check the full manuscript for this error] | Full text changed for this error. One additional found on line (163). |
| Line 140 –‘Only group A 141 from the study of Castiglione et al.[42] met the inclusion criteria for this review’ – does this meant that the same procedure was followed as the Deal study? Please be explicit about this. | Rephrased to be more explicit. It is only group A of Castiglione et al. and not Deal et al. Line: 146 |
| Line 152– error ‘therefor’ | Adjusted to “therefore.” Line: 157 |
| Line 153 – ‘We judged the deviations from THE intended intervention to be high..’ | The text has been amended. Line: 158 |
| Line 154 ‘there was insufficient assignment to the intervention’ …please clarify what this means - you mean the groups were imbalanced? | We have elaborated further in the text upon this issue. Line: 160-161 |
| Line 158 - write out ‘SPQM’ in full please | Has been adjusted to “short portable mental status questionnaire.” Line: 164-165 |
| Line 161 ‘ low risk in three STUDIES [30-32] and some concerns in one STUDY [29], | We changed the text accordingly. Line: 167-168 |
| Line 460 ‘ Lancet Commissions on Dementia’ please use the correct title for this paper and reference appropriately. | We changed this to the correct title for this paper and the reference has been adjusted. Line: 498-499 |

| **Reviewer 3** |  |
| --- | --- |
| This paper systematically evaluates and synthesises data from extant studies assessing the effects of hearing aid use on overall cognitive ability and specific domains of cognition. The paper addresses a major public health and clinical controversy and will be a timely and useful resource foe the field. Although it does not resolve the fundamental question whether hearing aid use can slow cognitive decline, the synthesis of Sanders et al. illustrates that the issues around the question are much more complex and nuanced than is often supposed and signposts directions that future research will need to take in order to resolve these issues. The focus on populations before onset of clinical dementia and the examination of specific cognitive domains are particular strengths of the study. I have a few comments intended to further strengthen the paper and contextualise it a little more clearly. | We thank the reviewer for their feedback and appreciate the comments raised.  We agree with the reviewer that by addressing the raised comments, we are better able to contextualise the paper. |
| 1. With respect to the information presented about the reviewed studies, it would be relevant to highlight hearing aid compliance wherever this was recorded – and if not recorded, that should be flagged as a limitation of the study. Hearing aids even when prescribed are frequently under-utilised and it cannot be assumed that the ‘treatment’ group actually was exposed to the treatment (or what ‘dose’ they actually received) without more detailed information. | We agree with the reviewer that hearing aid compliance data would be beneficial to the paper. It is surprising how often compliance is relatively low. We added this in our limitations. Line: 443-447 |
| 2. The authors might finesse a little more their prior hypotheses about the likely effects of hearing aid use on particular cognitive domains, and discuss the findings accordingly. For example, a priori it is not particularly surprising that episodic memory is not much affected by hearing aids. On the other hand, if hearing aid use has no impact on the decline of language skills that might be telling us something quite important. | This is an interesting point and something we have deliberated on by ourselves as well. There is a certain fear that by linking the hypotheses to the results without strong evidence that we might be infering or deducing certain connections that might not be there. On another note, episodic memory is affected by hearing aid use (as mentioned in the text) – episodic memory declined significantly with the addition of age, but this rate of decline, significantly decreased after starting hearing aid use (as shown by Maharani et al., 2018). |
| 3. Although cognitive function (and more particularly, cognitive test performance) is the main focus of this review it might be worth indicating explicitly that this is not, of course, the whole story in assessing the benefit of hearing aids – subjective measures of wellbeing and mood would also need to be taken into account as well as more specific impacts on interpersonal communication and relationships and social behaviour.  It might also be worth mentioning that there are other domains of cognitive function – such as social and emotional cognition – that may also be relevant but as yet have hardly been studied in relation to hearing aid use in older people. | This is an extremely valid point that the reviewer has brought up and we should expand on this more. We briefly touch upon this in the section “clinical applications and further research” but we have expanded upon it. Line: 481-482  Although social and cognitive function are not defined according to the criteria we used from the DSM, we do agree that these domains are important in considering to hearing aids. We therefore mentioned these domains shortly. Line: 496-498 |
| 4. In considering why the results are so mixed, the authors should consider a further explanation: that hearing aids do not address aspects of central hearing (auditory brain) function that may also be highly relevant to the link between hearing impairment and cognition. In fact, to the extent that central auditory factors interact with peripheral hearing loss, an inconsistent magnitude of benefit from peripheral amplification is exactly the pattern one might expect. This will only be resolved by:  i) the availability of adequate tests of central hearing and interventions (such as ‘smart’ hearing aids) that address central hearing, and their implementation in large scale trials of people at risk of dementia - at present, it is not clear which tests and/or devices will be optimal for this purpose though it is interesting to note that ‘smart’ hearing devices would be much easier to ‘blind’ (for the purpose of RCTs) than hearing aid use per se  ii) much more careful stratification of hearing function by proximity to the clinical onset of dementia – as it is possible that one reason hearing aids do not consistently ‘prevent’ dementia is that hearing loss is itself an early warning sign of dementia (i.e., auditory brain dysfunction)  I would recommend the authors broaden their Discussion to incorporate these considerations – as they rightly point out, the various putative mechanisms for linking hearing impairment to cognitive decline are by no means mutually exclusive.  In this regard, there are two recent relevant position papers presenting fresh hypotheses about the linkage that should be cited and discussed:  i) Griffiths TD, Lad M, Kumar S, Holmes E, McMurray B, Maguire EA, Billig AJ, Sedley W. How can hearing loss cause dementia? Neuron 2020; 108(3): 401-412. doi: 10.1016/j.neuron.2020.08.003.  ii) Johnson JCS, Marshall CR, Weil RS, Bamiou DE, Hardy CJD, Warren JD. Hearing and dementia: from ears to brain. Brain 2021; 144(2): 391-401. doi: | We thank you for bringing this to our attention. We find the newly proposed mechanism number 4, by Griffiths et al. (2020) very interesting and Johnson et al. (2021) make an important point about the oversimplification that happens in dichotomizing the ear and brain. We have expanded our discussion to try and incorporate these different arguments and hope this leads to a more balanced and well-rounded discussion. Lines: 393-431 |

| **Reviewer 4.** |  |
| --- | --- |
| Authors have systematically reviewed the existing literature to investigate the evidence for using hearing aids intervention as a treatment for further delaying or arresting further cognitive decline. Well-designed study. Appropriate tools have been used for the data analysis. A thorough assessment has been conducted on risk and bias of the studies. Very well-written manuscript. Authors have provided good directions for future research. | We thank the reviewer for this positive outlook towards our study. We realize that there is still a lot of unknown in this subject, but hope that this paper can provide insights into new perspectives a foundation for further research. |
