# Supplementary material for: The effect of hearing aids on cognitive function: A systematic review
Source: PLoS One. 2021 Dec 31;16(12):e0261207. doi: 10.1371/journal.pone.0261207 (PMC8719768; doi:10.1371/journal.pone.0261207)
Supplement: Attachment — Submitted filename: Response to Reviewers_IS.docx [file pone.0261207.s005.docx]

Dear Editor,

We would like to thank you for the chance to resubmit the manuscript ‘The effect of hearing aids on cognitive function: a systematic review’. Our response to the review comments can be find below.

Thank you for considering our manuscript for publication.

Sincerely,

On behalf of the author-team,

I. Stegeman

| **Review comment** | **Response** |
| --- | --- |
|  |  |
| line 117-119 DSM-V does not include general intelligence but does include social cognition as a cognitive domain. Please rephrase. (Similarly, on line 156-8, if the papers did not include social cognition then not all cognitive domains were covered). | Thank you for bringing this to our attention. Rectified. Line: 109-113 and 151. |
| line 119. THESE data were portrayed... | We changed the text accordingly. Line: 113 |
| line 172. The sentence started "We judged the measurement..." does not seem to make sense. | Thank you for hightlighting this unclarity. The text has been refrased. Line: 166-171 |
| line 177. was REPORTED numerically. | We changed the text accordingly. Line: 173 |
| line 424. medial temporal LOBE | We changed the text accordingly. Line: 418 |
| line 440. spell out PTA | Has been adjusted to “Pure tone average” Line: 433 |
| line 462. ...UNDERUTILIZE the hearing aids and THEREFORE... | We changed the text accordingly. Line: 453 |
| line 477. defined AS a HETEROGENEOUS clinical syndrome | We changed the text accordingly. Line: 467 |
